# Non-specific effects of inactivated *Mycobacterium bovis* oral and parenteral treatment in a rabbit scabies model

**DOI:** 10.1186/s13567-024-01293-y

**Published:** 2024-03-26

**Authors:** Rosa Casais, Natalia Iglesias, Iker A. Sevilla, Joseba M. Garrido, Ana Balseiro, Mercedes Dominguez, Ramon A. Juste

**Affiliations:** 1 Area de Sanidad Animal, SERIDA, 33394 Gijon, Asturias Spain; 2 Departamento de Sanidad Animal, NEIKER-BRTA, 48160 Derio, Bizkaia Spain; 3 Departamento de Sanidad Animal, Facultad de Veterinaria, Universidad de León, 24071 León, León Spain; 4 Unidad de Inmunología Microbiana, Centro Nacional de Microbiología, Instituto de Salud Carlos III, 28220 Majadahonda, Madrid Spain; 5 NySA Group, SERIDA, 33300 Villaviciosa, Asturias Spain

**Keywords:** *Sarcoptes scabiei*, *Mycobacterium bovis*, trained immunity, scabies, tuberculosis, serology, killed vaccine

## Abstract

Tuberculosis BCG vaccination induced non-specific protective effects in humans led to postulate the concept of trained immunity (TRAIM) as an innate type of immune mechanism that triggered by a pathogen, protects against others. Killed vaccines have been considered not to be effective. However, field efficacy of a commercial vaccine against paratuberculosis, as well as of a recently developed *M. bovis* heat-inactivated vaccine (HIMB) prompted to test whether it could also induce TRAIM. To this, we used a sarcoptic mange rabbit model. Twenty-four weaned rabbits were treated orally or subcutaneously with a suspension of either HIMB (10^7^ UFC) or placebo. Eighty-four days later the animals were challenged with approximately 5000 *S. scabiei* mites on the left hind limb. Skin lesion extension was measured every 2 weeks until 92 days post-infection (dpi). Two animals were killed at 77 dpi because of extensive skin damage. The rest were euthanized and necropsied and the lesion area and the mite burden per squared cm were estimated. Specific humoral immune responses to *S. scabiei* and to *M. bovis* were investigated with the corresponding specific ELISA tests. Subcutaneously and orally HIMB vaccinated animals compared with placebo showed reduced lesion scores (up to 74% and 62%, respectively) and mite counts (−170% and 39%, respectively). This, together with a significant positive correlation (r = 0.6276, *p* = 0.0031) between tuberculosis-specific antibodies and mite count at 92 dpi supported the hypothesis of non-specific effects of killed mycobacterial vaccination. Further research is needed to better understand this mechanism to maximize cross protection.

## Introduction

Non-specific effects (NSE) of vaccination have been reported since BCG (vaccine Bilié de Calmette et Guérin) vaccination became widely applied in some countries or regions, as a decrease of mortality beyond what could be expected from prevention of specific mortality caused by tuberculosis was observed [1, 2]. These NSE have also been noticed with other vaccines, like polio and measles [3, 4]. However, negative effects in females have somewhat blurred the overall effect [5] and driven a controversy that even though pointing to confirmation, has still not resulted in a recovery of BCG vaccination in developed countries or a general World Health Organization (WHO) recommendation on taking advantage of these NSE. The extraordinary therapeutic effect of active immunization against some forms of cancer [6] and the enunciation of a theory on training immunity [7, 8] that support these effects have further underlined the interest of confirming them. As a consequence, literature on the subject has started blooming. In veterinary medicine, the NSE effects have been longtime observed but mostly in a production perspective that had not driven an interest in the medical grounds until very recently. Therefore, the NSE inducers, broadly described as carriers of pathogen-associated molecular pattern (PAMP) [1, 7], have been longtime in use as diet supplements to improve feed conversion and to reduce antibiotic use [1, 9]. Although there are growing studies on the physiological mechanisms and metabolic pathways involved [10, 11], given the evolutionary primitive character of the phenomena that already appears in vegetals [12], it seems that it all orbits around the training of phagocytic cells for enhanced performance on secondary pathogen exposure [13].

The slow acceptance of a primitive trained immunity (TRAIM) at the phagocytic level, despite the huge potential of these mechanisms, can only be explained by the lack of experimental confirmation of phenomena observed in weaker non-planned epidemiological studies. Veterinary medicine has much to offer in this field, since it makes it easier to experimentally confirm in the target species the observations made in daily practice. The demonstration of the NSE induced by HIMB in different biological taxons (virus, bacteria, protozoa, arthropods) [14–17] prompted the use of a rabbit scabies model already reported by our group [18, 19] to produce further experimental evidence on these effects. Scabies is a disease caused by a microscopic mite, *Sarcoptes scabiei*, that affects different species, including humans, producing a severe dermatitis. We thought that it could shed light on the effects of two treatment variables that are critical to induce an immune response: route (oral and subcutaneous) and antigen (heat inactivated *Mycobacterium bovis* (HIMB) and placebo).

## Materials and methods

### Treatments and mite challenge with *S. scabiei*

**Figure 1:**
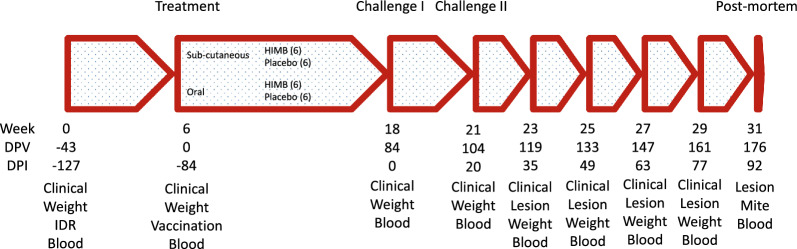
**Groups and time course of the experiment.** Control points in weeks from reception of rabbits and in days post-vaccination (DPV) and post-infection (DPI). Clinical: Observation and measurement of lesions; Weight: Rabbit individual weighting; IDR: Intradermal test; Blood: Blood extraction and processing; Lesion: Lesion area measurement; Mite: Mite count.

Twenty-five specific pathogen-free and scabies-free female New Zealand White 3-month-old rabbits were purchased from an authorized breeder (Granja San Bernardo, Navarra, Spain). Animals were tattooed in their ear for identification, housed in individual cages and allowed a 2-week acclimatization period. Rabbits were randomly allocated into 4 groups of six to receive a treatment (Figure 1). At the time of the first infection, one rabbit was infected and left without any treatment as a donor (DonNil) of the *S. scabiei* mites for inoculum maintenance at SERIDA. Treatments consisted of:

- Group SC-HIMB (rabbits 1–6): 0.5 mL of a suspension containing 0.25 mL of heat inactivated *M. bovis* (10^7^ UFC) (HIMB) in PBS and 0.25 mL of Montanide 50 adjuvant. Each animal was inoculated by a subcutaneous (SC) injection in the scapular region with a 21G needle using an insulin syringe.
- Group SC-Plbo (rabbits 7–12): 0.5 mL of a placebo with *M. bovis* suspension replaced by PBS (0.25 mL) and same amount of adjuvant (0.25 mL) administered in the same way as group A.
- Group OR-HIMB (rabbits 13–18): 1.5 mL of a suspension containing 1 mL of heat inactivated *M. bovis* (10^7^ UFC) (HIMB), 0.5 mL of extra virgin olive oil and 0.3 g of finely milled wheat bran given by the oral route with a 5 mL syringe without a needle.
- Group OR-Plbo (rabbits 19–24): 1.5 mL of placebo with *M. bovis* suspension replaced by 1.5 mL of PBS, 0.5 mL of extra virgin olive oil and 0.3 g of finely milled wheat bran delivered by the oral route with a 5 mL syringe without a needle.

On day 0, 14 days after rabbits’ arrival to SERIDA facilities, all animals were weighted and a sample of blood was taken from the auricular vein. In addition, they were submitted to a standard comparative intradermal tuberculin test to verify that they had no mycobacterial reactivity and as a possible priming before vaccination. Forty-three days later [84 days before challenge (−84 dpi)], the four experimental groups were treated as indicated above. The formation of nodules in subcutaneously inoculated animals was monitored 1 week and 1 month after treatment. Eighty-four days later all animals were submitted to a challenge by means of a dressing placed on the left hind limb (foot area) for 24 h carrying donor skin fragments containing approximately 2013 *S. scabiei* mites [0 days post-infection (dpi)]. Since animals removed the dressing during the first day, a second challenge was carried out 3 weeks later, at 20 dpi by means of a new dressing carrying approximately 5489 *S. scabiei* mites. The estimation of the number of mites per gr of skin fragments was carried out as previously described [18]. The time course of the experiment is shown in Figure 1.

### Clinical evaluation and mite density

**Figure 2:**
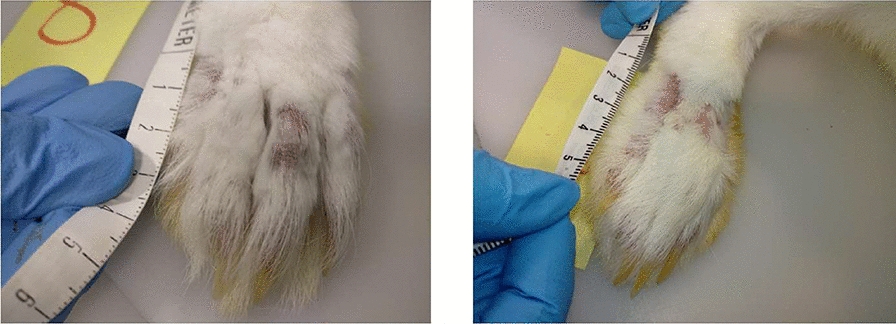
**Type of lesion and area measuring.** Lesions were mainly located in the inoculation areas and around the nails and consisted in hair loss and parakeratotic crusts. A measuring tape was used to estimate affected area width and length.

Animals were observed daily when cleaning, feeding, and watering. Body condition was assessed by measuring the changes in body weight, which was recorded at −127, −84, 0, 20, 35, 49, 63, 77 and 92 dpi. To summarize the net effects of *S. scabiei* on weight, the variable weight loss was created as the reduction of weight between the maximum recorded weight and the weight at the last control. Skin lesions were assessed for their extension every 2 weeks from 0 to 92 dpi. The lesion areas were photographed and measured using a flexible ruler according to Casais et al. [18] (Figure 2).

On day 77 post-infection, two animals (rabbits 17 of OR-HIMB and 21 of OR-Plbo) were euthanized by intravenous injection of pentobarbital (Dolethal ®, 100 mg per kg of body weight) after sedation with Ketamine at 5 mg/Kg and Xylazine 1 mg/Kg live weight given intravenously because of poor clinical condition. The remaining rabbits were euthanized at 92 dpi. After euthanasia, a left hind limb fragment of skin was taken from the lesioned area and stored at −20 °C. For mite counts, we followed the protocol described by Mumcuoglu et al. [20], which allows approximately 88% recovery of *S. scabiei* mites. Briefly, 4 cm^2^ of skin were suspended in 4 mL of 10% KOH solution containing 1% Tween 80, incubated for 18 ± 2 h at 45 °C, the material was then agitated for 2–3 min with a vortex and centrifuged at 500 × *g* for 15 min. Then, the supernatant was decanted to 1 cm above the pellet (about 2.5 mL were removed), which was suspended in the residual liquid, then 70% ethanol was added to get a final volume of 2 mL. Ten replicates of 25 μL digested suspension (a total of 250 μL) per rabbit were observed under the stereomicroscope and the total number of mites per skin cm^2^ was estimated.

### Immune response

In order to assess the humoral immune response (IgG) to *S. scabiei* infestation, blood samples were collected from the marginal ear vein of the rabbits at dpi −127, −84 (treatment), 0 (first challenge), 20 (second challenge) and every 2 weeks afterwards (dpi 35, 49, 63, 77 and 92) and stored at −20 °C. Anti-*S. scabiei* serum antibody levels were analyzed using an in house enzyme-linked immunosorbent assay (ELISca) based on the recombinant antigen Ssλ20ΔB3 [21, 22].

Specific response to *M. bovis* was confirmed with two different specific antibody ELISA tests. One was an in-house ELISA protocol based on p22 antigen performed as previously reported (ELIp22) [23, 24], and the other a commercial multispecies ELISA (INgezim Tuberculosis DR, Eurofins Technologies, Madrid, Spain) performed according to the supplier instructions (ELIMS).

Unstimulated blood gamma-interferon (γ-IFN) levels were measured at 6 time points (−127, −84, 0, 35, 49 and 92 dpi) as a proxy for systemic ongoing inflammatory responses in the experimental animals.

### Statistical analysis

Individual weight (IW), weight loss, ELISA indices, γ-IFN blood level, lesion surface and mite counts were submitted to the ANOVA analysis with jamovi [25–27]. Independent variables were route of administration of the treatment, type of treatment and dpi. Pre-planned paired (vaccinated/placebo, Oral/SC) group means were used for comparisons with the Student t pairwise tests of least square means. After observing the pattern of lesions and mite counts, it was noticed that the OR-Plbo group had higher values than any of the other treated groups and that these were more similar to those observed in the untreated donor rabbit (DonNil). Merging OR-Plbo with DonNil yielded a much lower standard deviation than for any of the other groups. Therefore, a post-hoc analysis was also carried out comparing all the other three groups to this combined one (PlaNil).

**Figure 3:**
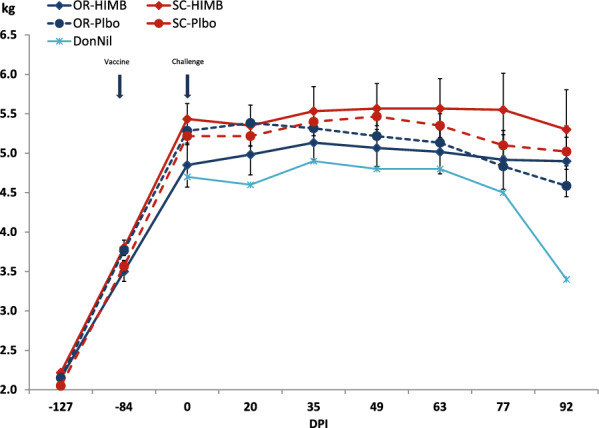
**Mean group bodyweight evolution during the experiment.** OR-HIMB: HIMB treatment by the oral route; SC-HIMB: HIMB treatment by the subcutaneous route. OR-Plbo: Placebo by the oral route; SC-Plbo: Placebo by the subcutaneous route. DonNil: Donor with no treatment.

Some treatment levels seemed to induce a reduction of variability, but most groups, including controls, showed a large variability that impaired useful discrimination between group responses. In particular, there were individual records that fell far away from the group mean. Therefore, in an attempt to rule out the fuzziness caused by these “outliers”, trimmed or truncated mean analysis was used to compare the main effects of each treatment against the control [28]. To this end, values that fell beyond the 95% confidence interval of the group mean and the overall standard deviation were discarded. As shown in Figure 3 for the case of weight loss, R^2^ increased by 116.7% and model significance rose from *p* = 0.1711 to *p* = 0.0198 (Figure 2).

To more easily assess the magnitude of the effect of the treatments, Cohen’s d and percent reduction were calculated and reported for the more relevant variables and time points comparisons. Correlations between variables were tested both for the whole dataset and for each treatment group separately [25, 27, 29]. Since the experimental groups were of small size, no specific statistical significance threshold was established, but probabilities were calculated and reported for all relevant hypotheses.

Also, to get some measure of the treatment effect on the outcome balance between pathology and infection, a lesion to pathogen relative reduction rate for the post-hoc groups was calculated. It was the quotient between the reductions of lesion area and mite count taking the PlaNil as the reduction reference value: (lesion reduction-mite reduction)/lesion reduction PlaNil. It was thought to be useful to better appraise the SC-HIMB group results where the mite count increase created a misleading negative reduction ratio.

Analysis of correlation was applied to the overall data to test associations between clinical, immune and pathological variables at different time points and also at the end of the experiment group-by-group to see whether these associations changed according to the immunization treatment. Both Pearson and Spearman indices were calculated to catch all types of quantitative associations.

## Results

### Impact of HIMB treatment/vaccination on weight, lesion area and mite burden

According to the age of the experimental animals, their weight increased up to 0 dpi and stabilized at around 5 kg for the rest of the experiment (Figure 3). No overall substantial differences in weight were observed between groups. The only overall significant main effect, apart from the normal effect of time on growth (*p* < 0.0001) which corresponded to lower weights up to 0 dpi, was the route of administration (*p* = 0.0013). This was related to an interaction with the type of treatment (*p* = 0.0272). The oral route was associated with a significantly global lower weight than the subcutaneous route (*p* = 0.0013). However, although the orally treated group weight mean was consistently lower than that of the subcutaneous at every time point but at −127 dpi, the overall weight difference between both groups was only marginally statistically significant at 77 and 92 dpi (*p* = 0.0527 and *p* = 0.0712, respectively) when the effects/lesion areas of infestation were more severe. The type of treatment did not show a significant global (*p* = 0.5764) or individual effect at each time point (*p* > 0.2010) on weight comparisons, however, there was a strong interaction (*p* = 0.0272) with route that indicated that HIMB treatment did not affect weight when given by the orally route (*p* = 0.2400, d = −0.2269, reduction 3.7%), but had a mild negative effect when given by the subcutaneous route (*p* = 0.0503, d = 0.3792, reduction −3.8%). The post-hoc analysis comparing with the PlaNil group indicated a significant difference in favor of the SC-HIMB group (*p* = 0.0009, d = −0.6330, increase 5.8%). This overall difference was mostly due to the difference at the two last time points. All the groups were heavier, but, especially, the SC-HIMB that was 16.0% and 20.0% heavier than the PlaNil at 77 and 92 dpi (*p* = 0.0162, d = −1.3494 and *p* = 0.0056, d = −1.5605, respectively). The largest difference in weight occurred at the 0 dpi just before challenge, when the OR-HIMB mean was 0.35 kg lower than that of the PlaNil which showed a moderate size of effect (d = 0.6279) but was not significant (*p* = 0.2681).

**Figure 4:**
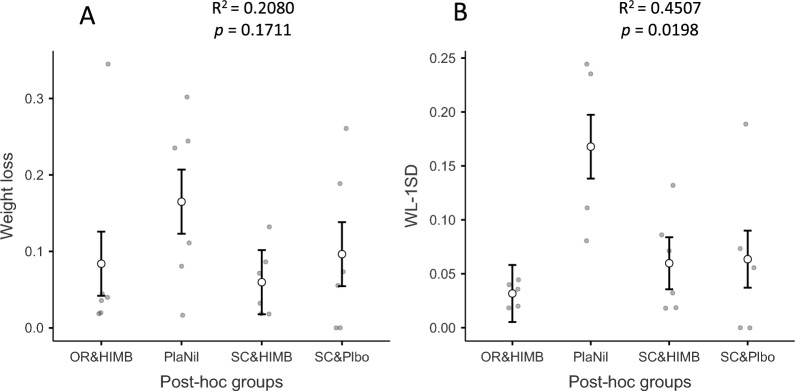
**Comparison of raw and trimmed weight loss analysis for post-hoc groups.** Panel A: Raw data. Panel B: Trimmed data. Error bars represent standard error of the mean. OR-HIMB: HIMB treatment by the oral route; SC-HIMB: HIMB treatment by the subcutaneous route. PlaNil: Placebo by the oral route and Donor with no treatment; SC-Plbo: Placebo by the subcutaneous route. WL-1SD: weight loss trimmed data at 1 standard deviation cut-off.

Regarding the post-challenge weight loss that somewhat summarizes the treatment effects on weight loss, it yielded a non-significant overall model (*p* = 0.3500), with the corresponding lack of effects for route (*p* = 0.2819), treatment (*p* = 0.1755) and their interaction (*p* = 0.6020). Restricting to the trimmed mean analysis, the model became statistically significant (*p* = 0.0198), but with only treatment (*p* = 0.0186) and its interaction (*p* = 0.0248) with route (*p* = 0.1727) showing an effect on weight loss. These effects were mainly due to the significant large weight loss (*p* = 0.0002) of the OR-Plbo group (19.79%) vs. the OR-HIMB (3.17%) while the SC-Plbo (6.5%) did not differ (*p* = 0.8940) from the SC-HIMB (5.98%). The trimmed mean analysis for the post-hoc grouping showed an overall significant effect (*p* = 0.0198) due to the differences of the PlaNil (combined OR-Plbo plus DonNil) group with the other three groups (SC-HIMB, *p* = 0.0120, d = 1.8280; OR-HIMB, *p* = 0.0034, d = −2.3023; SC-Plbo, *p* = 0.0182, d = 1.7642) (Figure 4).

**Figure 5:**
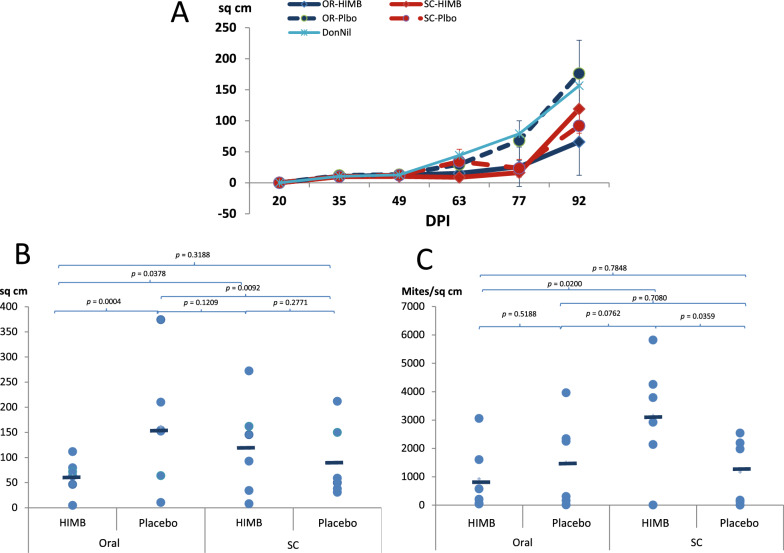
**Lesion area and mite burden.** **A** Mean group lesion area evolution. **B** Final mean group lesion area. **C** Final mean group mite count.

The protective effect of each treatment-vaccination in the rabbit-*S. scabiei var. cuniculi* animal model was more specifically assessed by measuring the affected areas over the course of infestation (Figure 5). No scabies lesions were observed at 20 dpi. However, all rabbits but one in group SC-Plbo developed scabies lesions by 35 dpi. Lesions, hair loss areas and parakeratotic crusts, were mainly located at the site of inoculation and around the nails. In some rabbits, lesions spread to other regions of the body such as nose and ears. Two animals, one from group SC-Plbo (placebo treatment, SC route) and one from group OR-HIMB (HIMB treatment, oral route) were euthanized ahead of schedule on 77 dpi because of poor clinical condition (rapid weight loss and severe evolution of skin lesions). The overall model for time, treatment and route was highly significant (R^2^ = 0.5055; *p* < 0.0001). At 35 dpi all groups showed about the same area of affected skin (oral route 10.46 cm^2^ vs. 11.92 cm^2^ and subcutaneous route 9.93 cm^2^ vs. 9.96 cm^2^; *p* = 0.9567 and *p* = 0.9991, HIMB vs. Placebo, respectively), the lesioned area moderately progressing by 49 dpi, and then very quickly until the last examination at 92 dpi (Figure 5A). At this moment, orally and subcutaneously HIMB-treated groups reached a mean affected surface of 66.02 cm^2^ and 119.1 cm^2^, while the oral and SC placebo groups had 176.03 cm^2^ and 91.8 cm^2^, respectively. Overall, for the oral route, the placebo control group showed mean lesion areas significantly larger than the HIMB treated group (*p* = 0.0004) while for the SC route no significant differences were observed between the placebo and the HIMB vaccinated groups (*p* = 0.2771). Therefore, there was as significant reduction in the lesion area associated to OR-HIMB treatment, while the subcutaneous treatment had just a slightly negative, non-significant effect. Limited significant differences between the OR-HIMB and the SC HIMB and SC Placebo groups (*p* = 0.0378, *p* = 0.3188, respectively), but larger differences of OR-Plbo with the SC-HIMB and SC-Plbo (*p* = 0.1209 and *p* = 0.0092, respectively) suggested that the SC placebo might had a reduction effect (Figure 5B). Trimmed mean analysis confirmed the raw analysis with a higher R^2^ of 0.8460. Likewise, post-hoc analysis comparing PlaNil with the other three groups simplified the analysis, indicating that all groups had smaller lesion areas than the PlaNil group, which in the post-hoc analysis pointed out significant effects by 77 dpi In this case, however, the SC-HIMB group showed a significant increase in the lesioned area compared to the OR-HIMB (*p* < 0.0001, d = −3.34) and even the SC-Plbo (*p* < 0.0001, d = 3.26).

**Figure 6:**
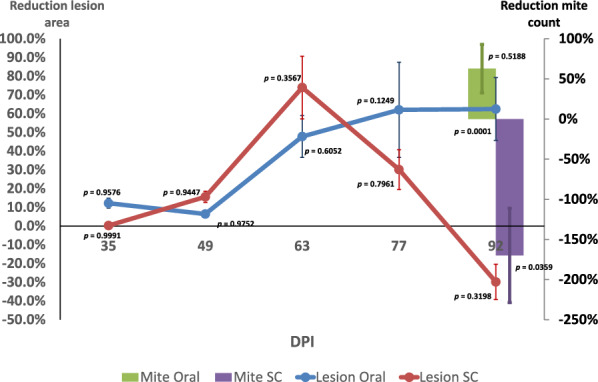
**Per cent reduction of lesion area and mite burden between HIMB and placebo groups.** Mite Oral: Mite count reduction percentage of orally treated HIMB group over oral Placebo; Mite SC: Mite count reduction percentage of subcutaneously treated HIMB group over subcutaneous Placebo; Lesion Oral: Lesion area reduction percentage of orally treated HIMB group over oral Placebo; Lesion SC: Lesion area reduction percentage of subcutaneously treated HIMB group over subcutaneous Placebo.

For the pre-planned model, these numbers represented an average global reduction of the lesions affected area with respect to the corresponding placebo of 56.5% and 4.1% for the oral and subcutaneous routes, respectively, in the whole study (Figure 6). While the subcutaneous group reached a maximum reduction of 74.0% by 63 dpi and then fell to an −32%, the orally treated group did not reach the maximum reduction until 77 dpi (62.1%) but maintained a similar reduction until 92 dpi (60.9%) (Figure 6).

The density of mites in the skin of all rabbits at 92 dpi was also analyzed as an indicator of the protective value of the different treatments (Figure 5C). In the rabbits treated by the oral route the mean number of mites per cm^2^ was higher in placebo (752.0 mites/cm^2^, ranging from 4 to mites 1981 mites/cm^2^) than in the HIMB treated group (462.2 mites/cm^2^, ranging from 20 to 1529 mites/cm^2^) although no significant differences were observed between the two groups (*p* = 0.5188). Unexpectedly, in animals treated by the SC route the mean number of mites per cm^2^ in rabbits treated with placebo (584.3 mites/cm^2^, ranging from 0 to 1270) was significantly lower than in rabbits treated with HIMB (1577.3 mites/cm^2^, ranging between 2 and 2910 mites/ cm^2^) (*p* = 0.0359). A great variability between individuals was observed within each group. These represented reductions of the mite density of 38.5% and −169.9% for the oral and SC routes (Figure 6), respectively. Comparing routes showed that the SC route favored a proliferation of mites when the treatment was HIMB (1577.3 vs. 462.2; *p* = 0.0200), while the oral did not (584.3 vs. 752.0; *p* = 0.7080).

Lesion to pathogen reduction rate the post-hoc analysis showed that in all cases the reduction was higher regarding lesions than mite burden with the lesion to pathogen ratio amounting to 1.32 (24.4%), −0.83 (360.9%) and 1.44 (30.4%) for OR-HIMB, SC-HIMB and SC-Plbo, respectively.

### Immune responses

#### S. scabiei var. cuniculi

**Figure 7:**
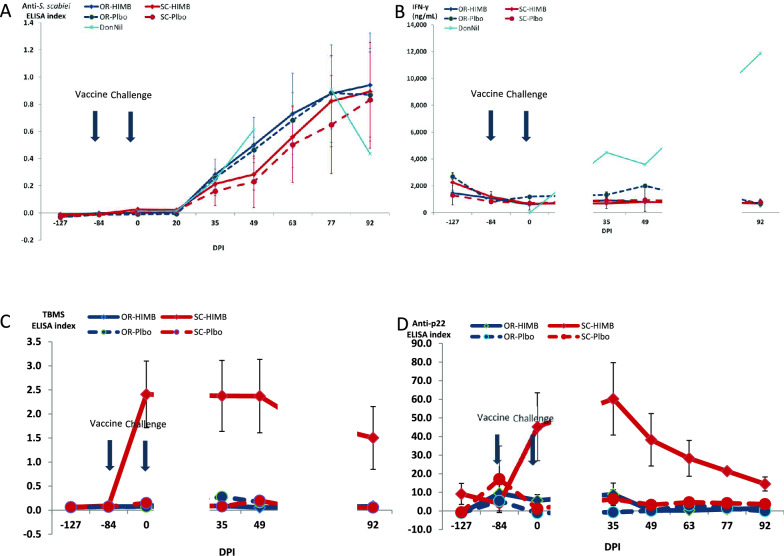
**Mean group immune response.** **A** Anti-*S. scabies* antibody response. **B** Non-specific IFNγ levels. **C** Anti-*Mycobacterium bovis* antibody mean group response in the Ingezym multispecies assay. **D** Anti-p22 antibody mean group response.

The humoral immune response (IgG) to *S. scabiei* var. *cuniculi* infestation was investigated using an in house designed ELISA. Time was the most significant main factor (*p* < 0.0001) affecting rabbit anti-*S. scabiei* antibody levels (Figure 7A), followed by the route of administration (*p* = 0.0038) and the interaction between the two (*p* = 0.1170). No differences between HIMB and Plbo groups were observed at any time point for either route of administration (Figure 7A). No significant differences were observed over time from day −127 pi to day 35 dpi for both HIMB groups, and then levels kept increasing and maintaining significance until the last sampling at 92 dpi. Regarding the route, no significant differences were observed during the first 5 time points. No differences between treatment groups were observed at any time point. However, route was important and by 49 dpi, there was a significant difference between oral and SC routes (*p* = 0.0020), that decreased by 63 dpi (*p* = 0.0161), 77 dpi (*p* = 0.0448) and disappeared by 92 dpi (*p* = 0.5454) (Figure 7A).

#### Tuberculosis

All animals were negative in the intradermal tuberculin test without any reactivity to the bovine or the avian tuberculin at the beginning of the study. All the SC-HIMB treated animals developed a specific humoral immune response that had reached a maximum on 0 dpi (84 days post-treatment) and that stayed high until 49 dpi and then started to slowly decrease until the last control (Figure 7C) according to the ELISA TBMS. The ELISA p22 showed some reactivity at −84 dpi, right after intradermal testing in all groups, but did not reach a maximum until 35 dpi. Afterwards, the reactivity decreased more sharply than in the ELISA TBMS until the last control (Figure 7D).

#### γ-IFN

Very low levels of γ-IFN were present at the first control (−127 dpi) that had decreased to negligible by the following control. Afterwards only the OR-Plbo showed very slight increases. However, the DonNil rabbit, which was only tested 4 times, showed a strong increasing trend that was still at a peak by 92 dpi (Figure 7B).

### Correlations

#### Overall associations

Weight was positively correlated with scabies antibody levels (r = 0.3491; *p* < 0.0001) and ELISA TBMS (r = 0.2867; *p* = 0.0003) and ELIp22 (r = 0213; *p* = 0.0080) and negatively with γ-IFN (r = −0.3040; *p* = 0.0003) in the overall analysis throughout the whole experiment. Taking all groups together and focusing on the outcome variables lesion area, mite count and weight loss, the most prominent correlations were those negative ones of lesion area at 49 to 77 dpi with weight from challenge (0 dpi) to end (92 dpi) (Table 1). Only the ELISA TBMS results at 49, 63 and 92 dpi showed some predictive value for mite count at the end of the study (r = 0.4716; *p* = 0.0200, r = 0.5367; *p* = 0.0069 and r = 0.6197; *p* = 0.0012). Lesion area did not show any significant correlation with immunological variables previous values. A clear association could be observed between lesion area from 49 dpi to the end and final weight loss, the stronger one being that with lesion area at 77 dpi (r = 0.80168; *p* < 0.0001). Considering all observations including the donor rabbit, the more synthetic clinical variable weight loss was positively correlated with lesion area (r = 0.5003; *p* = 0.0109) and mite count (r = 03747; *p* = 0.0649). Among immune variables only γ-IFN ELISA was positively correlated (r = 0.4271; *p* = 0.0332) with weight loss, with all the serological ones (scabies and TB) non-significantly negatively correlated with it.

**Table 1:** **Correlation matrix at 92 dpi**

	MitCou	LesAre	ELISca	IFNNil	ELIp22	ELIMS
LesAre						
Pearson’s r	0.6237***	–				
*p*-value	0.0009	–				
Spearman’s rho	0.6838***	–				
*p*-value	0.0002	–				
N	25	–				
ELISca						
Pearson’s r	−0.0068	0.3304***	–			
*p*-value	0.9742	0.0002	–			
Spearman’s rho	0.1831	0.3933***	–			
*p*-value	0.3794	< 0.0001	–			
N	25	124	–			
IFNNil						
Pearson’s r	0.2009	0.0706	−0.1017	–		
*p*-value	0.3355	0.5472	0.2204	–		
Spearman’s rho	0.2863	−0.1875	−0.1523	–		
*p*-value	0.1654	0.1073	0.0655	–		
N	25	75	147	–		
ELIp22						
Pearson’s r	0.4308	−0.1384	−0.0559	−0.1005	–	
*p*-value	0.0743	0.2067	0.4908	0.2553	–	
Spearman’s rho	0.257	−0.2191*	0.1353	−0.1638	–	
*p*-value	0.302	0.0439	0.0943	0.0625	–	
N	18	85	154	130	–	
ELIMS						
Pearson’s r	0.6276**	0.0444	0.0777	−0.1435	0.644***	–
*p*-value	0.0031	0.6793	0.3318	0.0982	< 0.0001	–
Spearman’s rho	0.3111	−0.2451*	−0.0634	−0.0194	0.4471***	–
*p*-value	0.1818	0.0206	0.4284	0.8243	< 0.0001	–
N	20	89	158	134	154	–
WeiLos						
Pearson’s r	0.3747	0.5003*	−0.1059	0.4271*	−0.2261	0.0203
*p*-value	0.0649	0.0109	0.6144	0.0332	0.367	0.9324
Spearman’s rho	0.5067**	0.6087**	0.0758	0.2102	0.0496	0.1214
*p*-value	0.0097	0.0012	0.7188	0.3133	0.8451	0.6102
N	25	25	25	25	18	20

MitCou: mite count; LesAre: Lesión area; ELISca: *S. scabiei* ELISA; IFNNil: γ-IFN ELISA without antigen stimulation; ELIp22: p22 tuberculosis ELISA; ELIMS: multispecies tuberculosis ELISA

*p < 0.05, **p < 0.01, ***p < 0.001

### Group associations

#### OR-HIMB

Weight at the start of the study (−127 dpi) was negatively correlated with lesion area at 35 dpi (r = 0.9179; *p* = 0.0098). This is not observed in any other group. In this group, other striking correlations are those positive ones between ELIMS at −84 dpi, at vaccination time, with lesion area at 35 dpi (r = 0.8365; *p* = 0.0379) and ELIMS at 49 dpi and lesion area at 63 dpi (r = 0.9532; *p* = 0.0032). Equally surprising are the correlations between IFNNil and lesion area, mite count and weight loss at 35 and 49 dpi. All are positive and strong (above r = 0.8000 an up to r = 0.9922; *p* < 0.0001). Weight loss was more strongly associated with lesion area at 77 dpi (r = 0.9660; *p* = 0.0017) than at any other time.

At the end of the experiment (92 dpi) weight loss was positively correlated with mite count (r = 0.8827; *p* = 0.0309), IFNNil (r = 0.8276; *p* = 0.0420) and ELIp22 (r = 0.8791; *p* = 0.0495) and mite count (r = 0.8527; *p* = 0.0309) but randomly and negatively with ELIMS (r = −0.1305; *p* = 0.8344) (Table 1).

#### OR-Placebo

Weight in this group was negatively correlated with lesion area between lesion area at 49 dpi and lesion area at 77 dpi since −84 dpi, that is, before vaccination and challenge until the end on 92 dpi. Correspondingly, weight loss was positively correlated with lesion area between 49 and 92 dpi. Regarding immunological variables, ELIMS at −84 dpi positively correlated with lesion area at 92 dpi (r = 0.8895; *p* = 0.0176), while at 35 dpi positively correlated with lesion area at 49 dpi (r = 0.8787; *p* = 0.0212) and lesion area at 77 dpi (r = 0.8909; *p* = 0.0172). IFNNil at −127 dpi correlated with lesion area at 35 dpi (0.8697; *p* = 0.0244), and ELIMS at 92 dpi negatively correlated with lesion area at 35 (r = −0.9756; *p* = 0.0244) and positively with lesion area at 63 (r = 0.9964; *p* = 0.0036), while the ELIMS at −127 dpi correlated with lesion area at 92 dpi (r = 0.8364; *p* = 0380). mite counts were negatively correlated with weight since −84 dpi to last control.

By the end of the experiment, there was a significant positive weight loss correlation with mite count that was weaker with lesion area and ELIMS. The strongest correlation was observed between scabies and ELIp22 (r = 0.9999; *p* = 0.0071). Strikingly five animals had a nearly perfect correlation between ELISca and lesion area (r = 0.9947; *p* = 0.00005). IFNNil correlated with lesion area (r = 0.9168; *p* = 0.0101) (Table 1).

#### SC-HIMB

This group is the one that showed the lower number of correlations. Weight negatively correlated with lesion area at 49 dpi since −84 dpi to 63 dpi. No correlations of ELIMS with any other variable were observed in this group which was the only one that showed strong ELIMS reactivity. Among immunological variables, ELISca at 63 dpi correlated with lesion area at 77 dpi (r = 0.8775; *p* = 0.0216) and 92 dpi (r = 0.9246; *p* = 00083). ELIp22 at final sampling negatively correlated with lesion area at 63 dpi. mite count was positively correlated with weight at −127 dpi. Weight loss significantly correlated negatively with ELIp22 (r = 0.8791; *p* = 0.0495) when taking all the observations.

At 92 dpi, weight loss negatively correlated with ELIp22 (r = −0.9115; *p* = 0.0312). Lesion area and mite count were positively correlated (r = 0.9015; *p* = 0.0141), and mite count and IFNNil were negatively correlated (r = −0.8630; 0.0269) (Table 1).

#### SC-Placebo

Weight negatively correlated with lesion area at 35 and 49 dpi and from then to the end. Humoral anti-*S. scabiei* response at the end of the experiment (77 [r = −0.8924; *p* = 0.0167] and 92 [r = −0.8906; *p* = 0.0173] dpi) negatively correlated with lesions at 35 dpi. Lesion area and ELISca negatively correlated at 49 dpi (r = −0.8698; *p* = 0.0243). Lesion area by 63 dpi correlated with ELISca at the final sampling (r = −0.9463; *p* = 0.0042). Only correlation with ELIMS was observed with lesion area 49 at −127 dpi (r = −0.8365; *p* = 00.379). This was the same time that correlation for lesion area and IFNNil was observed, however IFNNil at 49 dpi was correlated with lesion area at 63 dpi. Weight loss was positively correlated with lesion area at 63 and 77 dpi.

No significant correlations were found among any variable in this group at the end of the experiment (Table 1).

## Discussion

### General considerations

The results presented here depict a pattern that is consistent with an induction of non-specific effects by the administration of a *M. bovis* heat inactivated based-vaccine on both end-point main variables (lesion area and mite count) in the rabbit-*S. scabiei *var. cuniculi heterologous infestation model [2, 17, 30–34], thus supporting the induction of a TRAIM mechanism. This effect was statistically significant for lesions and for mite count even though the groups were small, despite the high variability in the individual immune responses and the fact that animals were submitted to a heavy challenge of two treatments, each with a high number of mites. Use of trimmed mean analysis strongly increased the statistical power of the experimental design even though the number of observations was substantially reduced for some treatment levels [28]. The objective of this analytical strategy was to focus on the main patterns activated by each treatment at the expense of leaving out individual behaviors that did not fit well. Since extremes on both sides of the mean values were left out, no bias should have been introduced. Actually, the groups that lost more observations were the placebo groups, this suggesting that HIMB treatment did have at least an overall homogenizing effect (Figure 4). This could be interpreted as vaccine treatments acting on a natural mechanism that can be triggered by unknown factors in un-treated individuals. Trimmed mean analysis, ignoring the influence of other factors, should allow to focus on the pre-planned factors in the current experimental design, which is HIMB treatment (and adjuvants in the case of SC-Plbo). We think this is valid for a first approach to study a primitive immune mechanism that evolved under complex combinations of natural pressures (genetics interacting with PAMP type route and timing) [1, 7, 12, 35], which is difficult to investigate due to its lack of specificity and likely involvement of many activation routes. Controlling all these pressures cases would require the step by step clearing strategy that we try to initiate here by focusing on the central effects and assuming we cannot yet explain the divergent cases [36] that would be the result of small individual differences in primary sensitization in the pre-experimental period [37].

### Clinical effects

Regarding weight loss the overall analysis only showed a standard (*p* < 0.05) significant difference between the SC-HIMB and the PlaNil (OR-Pblo-DonNil) group although the other groups also had experienced smaller losses (1.9 and 2.2 kg for SC-Plbo and OR-HIMB, respectively). Using the trimmed mean approach higher significant differences in weight loss were observed for all groups in the post-hoc grouping analysis reaching up to between three and sixfold those of the PlaNil. These results (smaller weight losses with respect to PlaNil) [37] can be interpreted as a clear effect of HIMB and even placebo (oil adjuvant by the subcutaneous route) treatment on this overall clinical disease variable [38, 39].

### Lesion area and mite count effects

An interesting effect is the different responses related to time and route. For instance, the effect on the lesion area. While the oral route was associated with a smaller decrease in affected area than the SC by 63 dpi, the effect increased in the following two samplings and stood stable by the end of the experiment. The SC treated group, however, reached a maximum percent decrease by 63 dpi, that quickly and steadily decreased until the last control at necropsy, when it became negative with respect of its placebo. This suggests that the beneficial effects of treatment over lesion development occur only within a narrow range of immune stimulation, easily becoming negative when stimulation exceeds a limit. It also warns on the timing of the optimal response regarding experimental designs because it seems possible that the desirable long-term effects cannot be seen with shorter experiment follow-ups. Additionally, it seems that the oral route might be a good delivery way [40, 41], thus supporting the beneficial effects of use of immune enhancers in the diet [1]. This is also consistent with a mechanism developed and operating in natural conditions by chance when the right conditions concur. More important in practical terms is that the effect is not related to specific sensitization given that no specific anti-*M. bovis* antibodies were detected in this group. The other consequence of this study is that a tolerizing effects [42, 43] could have been developed with worsening of the experimental animal’s clinical outcome in the SC-HIMB group, the one that more directly was exposed to the mycobacterial antigen because the parenteral route skips the natural environment/organism barrier. This worsening of the lesions outcome might be due to an exacerbating immune response that we have defined as divergent in badgers [36] and that has also been observed in other studies related to the type of trigger [44]. Anyway, this seems to be in contradiction with the observation that the TB antibody response was negatively correlated with the summary variable weight loss in the SC-HIMB group, while positively in the OR-HIMB group. Actually, as can be seen in Figure 4, individual results within each group might widely vary and, one OR-HIMB vaccinated animal had to be euthanized because of its bad clinical status. This suggests that even though a TRAIM beneficial overall effect is reached, the treatment has some risks at individual level due to immune response variability and other factors such a heavy challenge as the one applied in this experiment, where it was repeated with more than twice the dose 3 weeks after the first one for fear of poor challenge administration [39]. It can be speculated that a more natural, less intensive challenge would have either prevented mite colonization, or reduced subsequent severity. This would have allowed a longer follow up in which perhaps the negative effects could have been reversed. Route probably can be related to exposition or effective dose since it is well known that the oral route provides less antigenic stimulus than the parenteral one [45–49].

### Antigens and adjuvants

On the one hand, the SC route placebo that contains a non-specific adjuvant showed consistently lower lesion and mite burden than the OR-Plbo. Adjuvants are used because they enhance immune response against different antigens, therefore they can be considered as non-specific [37, 50, 51]. The experimental design assumed that the orally administered placebo that did not contain any immunoactive compound and showed the worse results regarding “protection”, had no effect. However, it seemed to modify the humoral response since anti*-S. scabiei* antibody levels were similar to the orally HIMB treated animals and significantly higher than those of SC treated or control groups. Also, there were some correlations of ELIMS results with clinical variables. In the γ-IFN analysis, the DonNil animal showed very high and increasing values at the end of the experiment that were not matched by the OR-Plbo group. Therefore, it seemed that this group might have experienced some benefit from the initial intradermal test exposition to mycobacterial antigens. This was not considered in the experimental design and therefore cannot be interpreted as a properly studied effect, but is suggestive; further experiments are needed to confirm or rule out that (1) the small IDR sensitization could have a non-specific effect, (2) treatment effects could have been even stronger if the IDT test had not been applied to the principal and (3) the TRAIM that lends a base to the effects approached in this study would be an extremely sensitive mechanism that can be triggered by many factors. In that sense, it is noteworthy to point out that the trimmed mean analysis can provide a useful weeding strategy for studying the innate TRAIM mechanisms while the relevant factors controlling its functions are being identified and experimentally controlled.

### Variable correlations

Overall correlation throughout the whole study that disappear when considering sampling by sampling, suggest that it is due to group interaction with time sequence, not by real dependence or common mechanisms. When separately considering the successive/consecutive time points it appears that lesion area was negatively correlated with weight. That could be explained as a deleterious effect of inflammatory lesions on general rabbit condition summarized in the animal’s weight following an interaction with treatment that starts at the day of challenge (0 dpi). *S. scabiei* ELISA at 77 dpi negative correlation with lesion area at 63 dpi could represent some success of specific anti-*S. scabiei* humoral response. However positive correlation of *S. scabiei* ELISA at 35 dpi with lesion area at 77 dpi might indicate that early reactivity might favor lesion development. This would be further supported by the positive correlation of multispecies ELISA with mite count at the end of the study suggesting that antibody response could be associated with pro-inflammatory effects [52]. Weight loss association with lesion area from 49 to 92 dpi, suggests that, indeed, the inflammatory processes causing lesions also influence weight loss [18, 19, 38, 39]. Positive association of IFNNil at −127 dpi with lesion area at 77 and of IFNNil at 00 dpi and 35 dpi with lesion area at 92 further support that view.

Since treating the data as group-aggregated results might combine treatment and individual effects, group by group analysis should allow to better identify individual mechanisms. Thus, starting with the OR-HIMB group, only the multispecies ELISA results at 49, 63 and 92 dpi showed a predictive value for mite count at the end of the study (r = 0.4716; *p* = 0.0200, r = 0.5367; *p* = 0.0069 and r = 0.6197; *p* = 0.0012). Lesion area did not show any significant correlation with immunological variables previous values. However, a clear association could be observed between lesion area from 49 dpi to the end and final weight loss, the stronger one being that with lesion area at 77 dpi which is the closer time point. Weight at −127 dpi correlates with lesion area at 35 dpi which suggests that general condition at arrival might have conditioned response to OR-HIMB treatment. That could be explained as PAMPs present in food microbiota and even vegetal diet components interacting with treatments [53–56]. This might be a strong limitation for studies on non-specific immunity that can be triggered by so many agents likely depending on time, route and dose. An observation with some practical value is that IFNNil at 35–75 dpi might have a broad predictive value of clinical outcome in terms of lesion area, mite count and weight loss.

Clinical variables weight and lesion area were the most frequently correlated variables in the OR-Plbo group, appearing especially relevant during the period 49 to 77 dpi (lesion area and weight at 63 dpi reached the highest correlation coefficient). Immunological variables did not seem to be very meaningful in this experiment since some of their correlations corresponded to the pre-experimental time point at −127 dpi (IFNNil, ELIMS), unless they were interpreted as pre-existing conditions modulating later pathological responses in line with the above-mentioned difficulty posed to the study of this type of immunity by natural non-specific sensitizations. The ELISca correlation between −84 dpi and lesion area at the end of the experiment and of 35 dpi and lesion area at 77 dpi could be interpreted as a weak response to IDR testing manifesting only after challenge, but lack of further time points correlations throws some doubts on any real meaning.

Regarding the SC-HIMB group, weight correlation since the beginning of the study right after intradermal testing with lesion area at 49 dpi might have heavily conditioned all the experiment results. Intradermal testing effect might have manifested as a weight change later related to lesion area extension and mite count. This would be similar to what was observed in OR-Plbo for lesion area, except that in this case the association was positive and anticipated for mite count. Interferon levels negatively correlated with mite count might reflect a failed mechanism of pathogen control based on IFNNil. ELISca response in SC-Plbo group could be explained as the result of the inflammatory changes seen as lesion area in previous controls. Early status regarding ELIMS and non-specific IFNNil at −127 dpi appeared to associate with lesion area by 35–49 dpi which were negatively correlated with weight. However, the best positive correlations between weight loss and lesion area occurred by the late samplings at 63 and 77 dpi.

A potentially interesting observation is that reduction in host response variables is higher, even much higher for the SC-HIMB group, than reduction in pathogen. This is reflected by the lesion to pathogen relative reduction rate although more confusedly by the absolute rate. This is consistent with the mechanisms involved in the response to the treatments used in this experiment being more protective for disease than for infection. That is, with having a limited effect in destroying or impairing pathogen proliferation but being highly efficient in restricting inflammatory changes triggered by them [57–59]. This is good news regarding non-specific protection against new diseases even though pathogen spread is not efficiently stopped. This might be of little value for pathogen eradication, but highly valuable for coexistence with pathogen without disease. The first is an important objective in veterinary medicine regarding new agents, even though it might favor spread of resistance or resilience for endemic diseases at a low cost. The second is more important in human medicine where presence or absence of a pathogen not causing disease is not relevant or might even be beneficial in order to keep immunological fitness at its best.

In conclusion, this study experimentally proofs the concept raised from field observations that mycobacterial vaccination or sensitization induces NSE and does it for the first time on a metazoan. This confirms the broad spectrum of the TRAIM mechanisms and indicates both a beneficial effect and a damaging one depending on route/dose and time with a series of interplays between immune, pathogen and clinical variables leading to different outcomes that need further research. Interestingly, lower doses seem to have more persistent beneficial effects, although these results require verification in larger experimental groups, with a longer follow up and training agent-pathogen specific interactions. Future experiments must address the time/dose/route issue, but also study the cellular mechanisms involved.

## Data Availability

Raw data are available upon request.
